# Supplementary material for: Cell Type-Specific Role of RNA Nuclease SMG6 in Neurogenesis
Source: Cells. 2021 Nov 30;10(12):3365. doi: 10.3390/cells10123365 (PMC8699217; doi:10.3390/cells10123365)
Supplement: Supplementary file 1 [file cells-10-03365-s001.zip › cells-1459367-Supplementary-Figures.pdf]

Supplementary Figures S1-S4

Guerra et al.

A

Genotype	E14.5 Number of isolated embryos	E14.5 Percentage of total (%)	Expected percentage (%)	E18.5 Number of isolated embryos	E18.5 Percentage of total (%)	Expected percentage (%)
Smg6 F/Δ; Nestin Cre +/+	5	21	25	18	27	25
Smg6 F/+; Nestin Cre +/+	8	33	25	17	25	25
Smg6 F/+; Nestin Cre Tg/+	4	17	25	20	30	25
Smg6 F/Δ; Nestin Cre Tg/+	7	29	25	12	18	25
Total	24	100	100	67	100	100

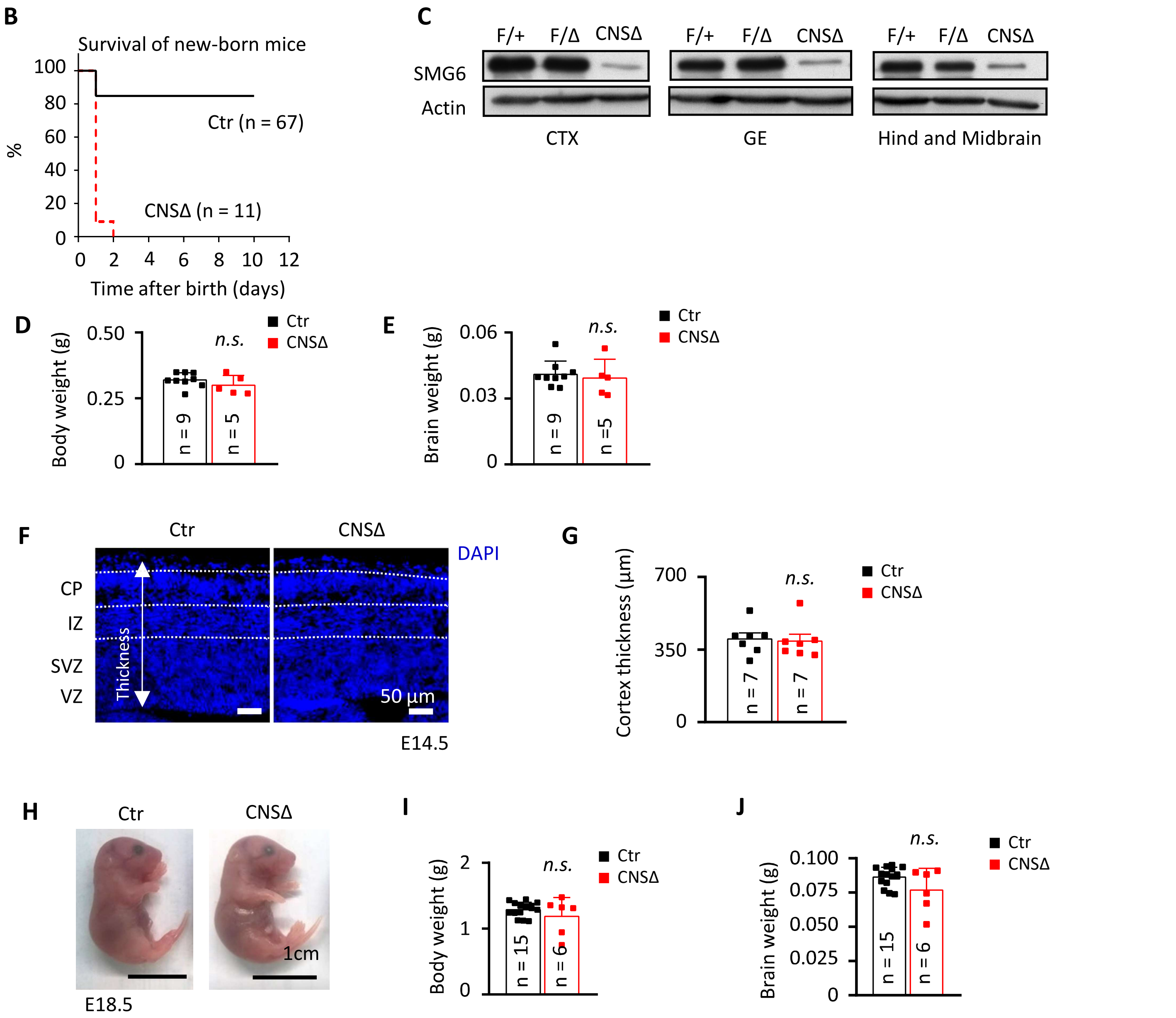

Figure S1. ***Smg6*** **deletion in all neuroprogenitors. (A)** Comparison of obtained and expected Mendelian ratios of E14.5 and E18.5 embryo genotypes from breeding *Smg6*$^{flox/flox}$ X *Smg6*$^{-/+}$;Nes-Cre^{tg}. **(B)** Survival of the *Smg6*-CNSΔ newborn mice in comparison to the controls (n: number of newborn mice per group). **(C)** Western blot analysis of SMG6 expression levels in CTX, GE and hind/midbrain at E18.5. Actin was used as a loading control. Quantifications of E14.5 embryo body **(D)** and brain weights **(E)**. **(F)** Comparison of DAPI stained CTX at E14.5, arrow indicates measurement of thickness quantified in **(G)**. Images and quantifications of the embryo body **(H,I)** and brain weights **(J)** at E18.5. For all graphs: n - number of embryos analysed. Error bars represent SEM. Statistics by unpaired Student`s *t*-test, except in **E** and **G** where the MWU was used, significance - n.s. > 0.05. VZ: ventricular zone, SVZ: subventricular zone, MZ: marginal zone.

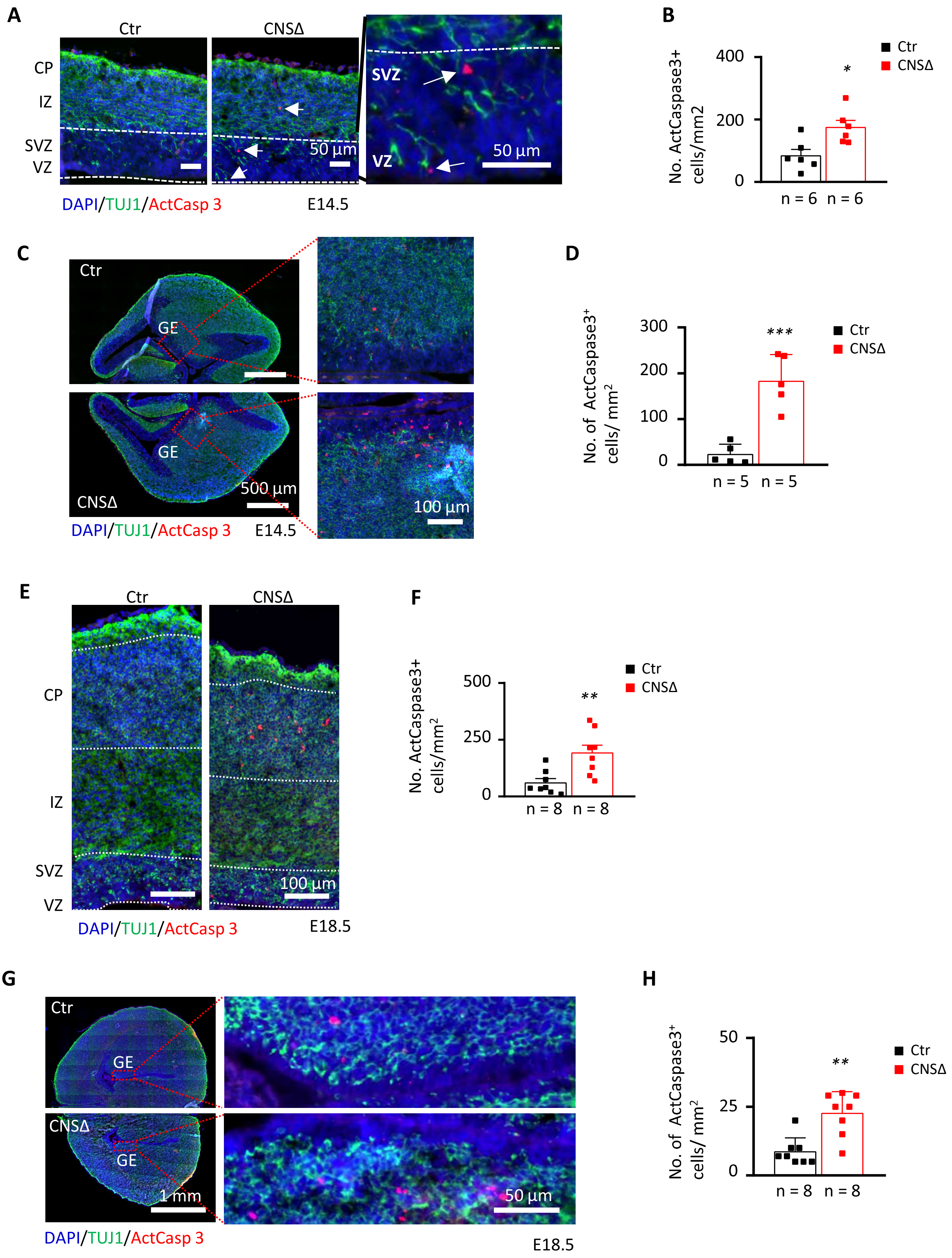

Figure S2. ***Smg6* deletion in the central nervous system cause Caspase3 dependent cell death in the cortex and ganglionic eminence.** **(A)** Active Caspase 3 and Tuj1 (Neurons) co-staining of E14.5 cortices with quantification of Act-Caspase3 positive cells shown in **(B)**. **(C,D)** Active Caspase 3 and Tuj1 (Neurons) co-staining of GE at E14.5 with quantification. **(E,F)** Images and quantification of Active Caspase 3 signals in CTX at E14.5 and **(G,H)** in GE at E18.5. For all graphs: n - number of embryos analysed. Error bars represent SEM. Statistics by unpaired Student`s *t*-test, except in **H** the MWU was used, significance - * <0.05, ** <0.01, *** <0.001. VZ: ventricular zone, SVZ: subventricular zone. DAPI counterstains cell nucleus.

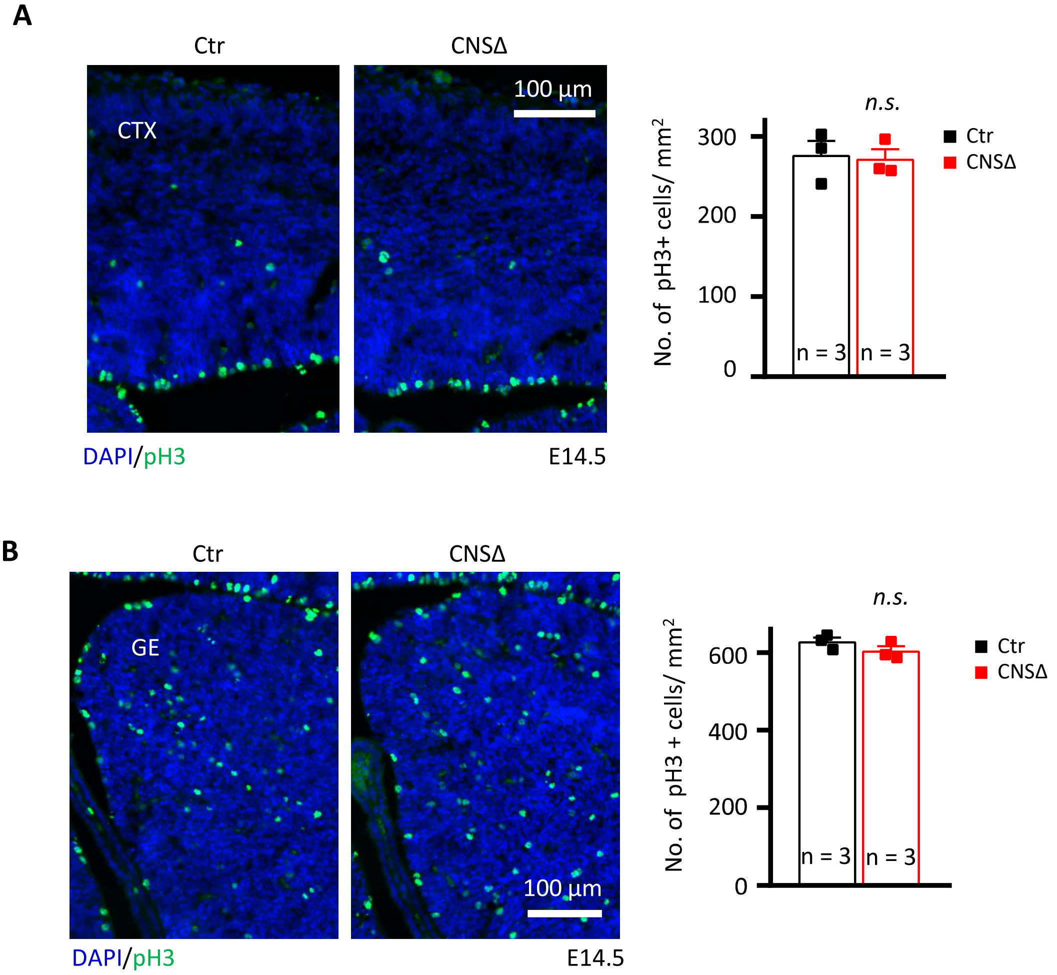

Figure S3. Proliferation of neuroprogenitors in the cortex and ganglionic eminence of E14.5 brains of indicated genotypes. Quantification of mitotic cells after immuno-staining of the CTX **(A)** and GE **(B)** using anti phospho-Histone H3 (Ser10) antibody (pH3). n - number of embryos analysed. Error bars represent SEM. Statistics by unpaired Student`s *t*-test, significance - n.s. > 0.05.

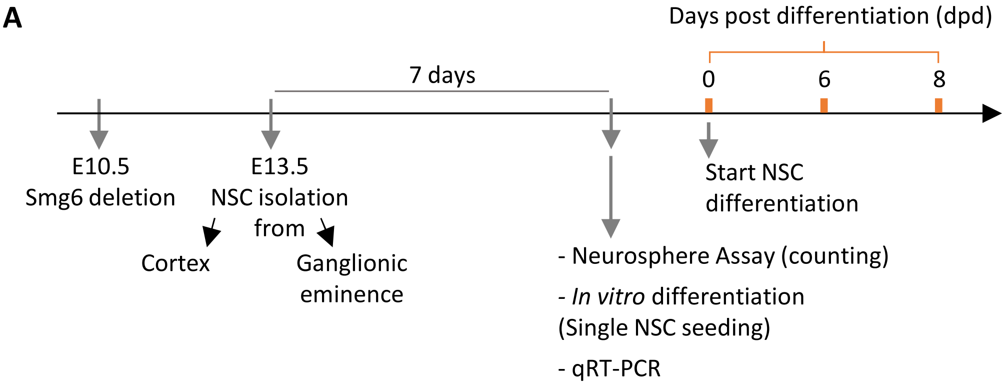

B

Genotype	Number of newborns	Percentage of total (%)	Expected percentage (%)
Smg6 F/F; Emx1 Cre +/+	14	21.2	25
Smg6 F/+; Emx1 Cre +/+	22	23.3	25
Smg6 F/+; Emx1 Cre Tg/+	14	21.2	25
Smg6 F/F; Emx1 Cre Tg/+	16	24.3	25
Total	66	100	100

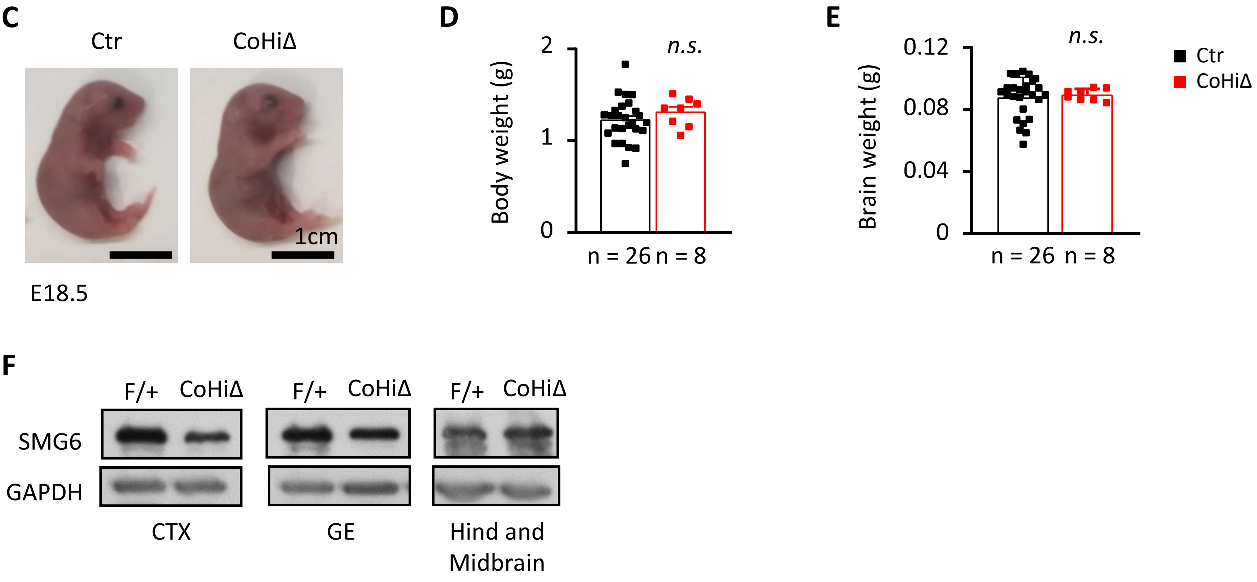

Figure S4. *Smg6* deletion only in cortex and hippocampus does not affect embryo development. (A) Experimental scheme used for the *in vitro* neurosphere assay, the *in vitro* differentiation and the qPCR analysis. **(B)** Comparison of obtained and expected Mendelian ratios of E18.5 embryo genotypes from breeding *Smg6*$^{flox/flox}$ X *Smg6*$^{flox/+}$; Emx1-Cre^{tg}. Body **(C,D)** and brain weights **(E)** in *Smg6*-CoHiΔ E18.5 embryos are normal. **(F)** Western blot of CTX, GE and hind/midbrain at E18.5 showing *Smg6* deletion in CTX but not in other parts of brain. Statistics by unpaired Student`s *t*-test in **D** and MWU in **E**, significance - n.s. > 0.05
